# Label‐free live cell imaging by Confocal Raman Microscopy identifies CHO host and producer cell lines

**DOI:** 10.1002/biot.201600037

**Published:** 2016-09-23

**Authors:** Batirtze Prats Mateu, Eva Harreither, Markus Schosserer, Verena Puxbaum, Elisabeth Gludovacz, Nicole Borth, Notburga Gierlinger, Johannes Grillari

**Affiliations:** ^1^ Institute of Physics and Materials SciencesBOKU University of Natural Resources and Life Sciences ViennaViennaAustria; ^2^ Department of BiotechnologyBOKU University of Natural Resources and Life Sciences ViennaViennaAustria; ^3^ ACIB Austrian Center of Industrial BiotechnologyGrazAustria

**Keywords:** Chinese hamster ovary cells, Label‐free detection, Raman microscopy, Single cell analysis

## Abstract

As a possible viable and non‐invasive method to identify high producing cells, Confocal Raman Microscopy was shown to be able to differentiate CHO host cell lines and derivative production clones. Cluster analysis of spectra and their derivatives was able to differentiate between different producer cell lines and a host, and also distinguished between an intracellular region of high lipid and protein content that in structure resembles the Endoplasmic Reticulum. This ability to identify the ER may be a major contributor to the identification of high producers. PCA enabled the discrimination even of host cell lines and their subclones with inherently higher production capacity. The method is thus a promising option that may contribute to early, non‐invasive identification of high potential candidates during cell line development and possibly could also be used for proof of identity of established production clones.

**Abbreviations**
CHO: Chinese hamster ovary
CRM: Confocal Raman Microscopy
ER: endoplasmic reticulum
FACS: fluorescence activated cell sorting
PDI: protein disulfide isomerase

See accompanying commentary by Katja Schenke‐Layland DOI 10.1002/biot.201600412

## 1 Introduction

Chinese hamster ovary (CHO) cells are the most abundantly used mammalian cells for the production of recombinant biopharmaceuticals 1. The successful development of producer cell lines is a long and strenuous procedure with hundreds to thousands of potential clones screened, tested and thoroughly characterized. Methods for cell line development rely on classical, dilution‐based plating as well as high throughput methods using robotics or fluorescence activated cell sorting (FACS) 2, 3 followed by detailed characterization of subclones. Moreover, testing the identity and stability of producer lines from master to working cell bank to process is complex and time consuming. Characterization methods typically require large numbers of cells (thus preventing early screening at microtiter scale), or are hampered by extensive, not always viable, sample preparation or labelling using antibodies and/or fluorophores. Thus, label‐free and viable analytical methods that work on the single cell level are of interest to support early selection of promising candidates.

Raman spectroscopy relies on the inelastic scattering of light when it interacts with matter. Changes in the polarizability of molecules cause an exchange of energy between the incident and the scattered light due to molecular vibrations (e.g. bond stretching). This energy shift is measured and the resulting Raman spectrum thus provides a ”molecular fingerprint“ with information about specific molecular bonds and their corresponding micro‐environment 4, 5. It has been widely used in the identification of chemical compounds in areas ranging from basic research (e.g. graphene characterization 6) to quality control 7 and chemical identification (e.g. detection of impurities such as 2,4‐dinitrotoluene vapor to locate landmines 6, 8). In CHO bioprocessing, Raman spectroscopy together with chemometrics 9, 10, 11 was proven useful for in situ monitoring of cell culture broth, in 2 to 5000 L bioprocesses 12, 13, 14. Still, information on a single‐cell‐basis, e.g. identity, productivity or morphological parameters such as organelle distribution or plasma membrane integrity, cannot be provided by such bulk spectroscopic methods.

Confocal Raman Microscopy (CRM), however, is a non‐invasive and label‐free imaging method that can be performed in situ on single live cells in a non‐destructive manner 15. The strength of Raman microscopy lies in the combination of high lateral resolution of about 300 nm (NA 1.4, λ_ex_ 532 nm) gained from the optical microscope and the sensitive chemical information derived from the Raman spectra 5, 16. The resulting Raman image is composed of thousands of spectra where each pixel represents the chemical profile for all Raman‐active components simultaneously. Previous studies in mammalian cells have already shown the potential of this powerful technique in discrimination of primary cells from established cell lines 17, 18, 19, characterization of stem cells and their susceptibility towards differentiation 20, 21 or identification of various stages of celI death 22.

The large amount of data generated in Raman imaging and the possible presence of overlapping bands in a spectrum require an unbiased way of unmixing the spectral information 23. CRM and multivariate data analysis approaches have been successfully combined to reveal non‐dominant features and small spectral variations 24, 25, 26.

In the current work, we have used CRM to distinguish different CHO host and producer cell lines in a label free manner, without prior sample preparation. Indeed, we show that subcellular structures that are enriched in specific chemical compounds like proteins, lipids, DNA or RNA and metal ions can be visualized by combining Raman spectroscopy with multivariate analysis using cluster analyses and PCA. Specifically a subcellular structure with high lipid and protein content, resembling the ER, was clearly distinguishable, whose size correlates to the production capacity of a cell line. Our data therefore suggest that CRM might be a valuable analytical tool for identification of cells with biotechnologically relevant characteristics during cell line development or engineering, but potentially also for quality control, proof of identity and stability of production cell lines.

## 2 Materials and methods

### 2.1 Cell culture and sample preparation

Host cell lines included CHO‐S (Invitrogen) and its subclone CHO‐S/4F11, CHO‐K1 (ECACC CCL‐61) and its subclones CHO‐K1/4F10 and 1D9. The subclones were isolated after multiple rounds of cell sorting for increased transient productivity 27. Producer cell lines were CHO‐S‐Humira, producing the human monoclonal antibody Adalimumab and CHO‐K1‐hDAO expressing the copper‐containing human diamine oxidase 28. All cell lines were cultured in CD‐CHO medium (Gibco^®^), supplemented with 8 mM L‐glutamine (Merck Millipore) and 0.2% Anti‐Clumping Agent (Gibco^®^) in shaker flasks at 140 rpm, 37°C, 7% CO_2_. CHO‐K1‐hDAO medium was in addition supplemented with 10 µM CuSO_4_ (Sigma Aldrich) and 10 µg/mL Blasticidin‐S‐HCl (Life Technologies). Viability was measured by Trypan Blue and better than 95% during all experiments.

For CRM analysis, 1.5 mL cell suspension were centrifuged at 170 × *g* for 7 min and resuspended in fresh, pre‐warmed growth medium. Cell suspension (15 µL) was applied to a glass microscope slide, covered with a #1.5 glass cover slip (Menzel‐Glaeser, Germany) and sealed with nail polish.

### 2.2 Confocal Raman microscopy

Raman spectra of cells were acquired using an upright Confocal Raman microscope (alpha300RA, WITec GmbH, Germany) with a 100× oil immersion objective (NA 1.4) (Carl Zeiss, Germany). The sample was excited with a linear polarized (0°) coherent compass sapphire green laser λ_ex_ = 532 nm (WITec, Germany). The scattered Raman signal was detected with an optic multifiber (50 nm diameter) directed to a spectrometer UHTS 300 (WITec, Germany) (600 g mm^−1^ grating) and finally to the CCD camera (DU401 BV, Andor, North Ireland). Control Four (WITec, Germany) acquisition software was used for the Raman imaging set up. Spectra were recorded in 1 µm X/Y steps for all samples with the exception of cells used for viable cell analysis, where spectra were acquired every 500 nm. The laser power was set to 30 mW and an integration time of 0.5 s was chosen to ensure fast mapping and to avoid cell damage.

### 2.3 Spectral pre‐processing and data analysis

All spectra were subjected to cosmic ray removal using a cosmic ray detection algorithm (filter size of two spectral pixels with a sensitivity indicated by a dynamic factor of eight) in the software Witec Project Plus 4.0.

#### 2.3.1 Cluster analysis of cell averages: CHO‐K1 and protein producers

**Figure 1:**
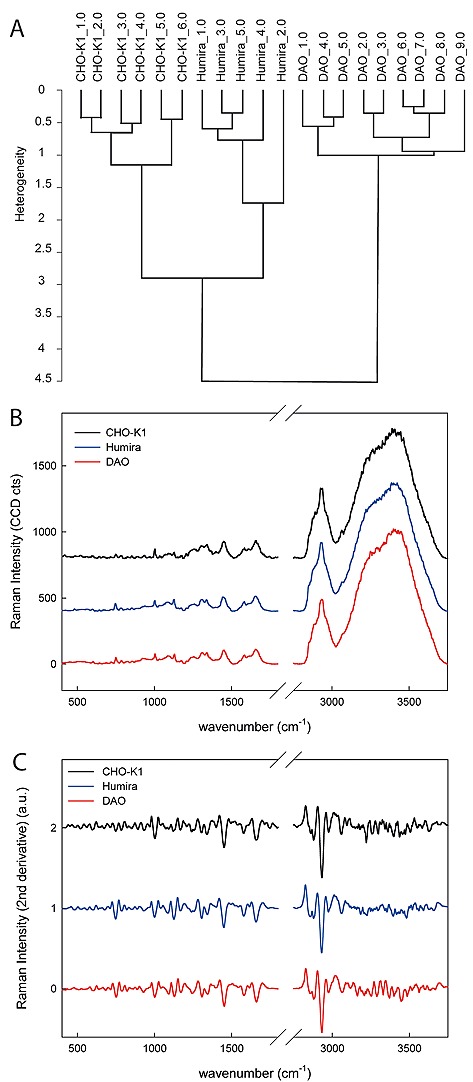
Raman microscopy spectra separate CHO host and producer cell lines. (**A**) Cluster analysis based on the spectral region 403–3750 cm^−1^ of the host line CHO‐K1 (*n* = 6 cells) and the two protein producers CHO‐K1‐hDAO (*n* = 9) and CHO‐S‐Humira (*n* = 5). All individual cells of each cell line were separated into different clusters indicating sufficient differences in their spectral characteristics. (**B**) Average spectra in the measured range of each cell line. (**C**) Second derivative spectra used for cluster analysis (baseline corrected and second derivative with 17 smoothing points). For (**B**) and (**C**) the spectra are stacked on the y‐axis to allow for clear discrimination.

The Raman spectra of the image scans of the host cell line CHO‐K1 and protein producers CHO‐K1‐hDAO and CHO‐S‐Humira were averaged using a mask filter in order to use only the areas comprising the cells. The average spectra of each cell were baseline corrected and derived (second derivative, 17 smoothing points) prior to cluster analysis (wave number region 403–3750 cm^−1^), based on the Euclidean distance and follows Ward's minimum variance algorithm: the distance between neighbors is given by heterogeneity (OPUS software, Bruker Optic GmbH, Germany). Spectra gathered in the same cluster were used to calculate the average spectra shown in Fig. 1B and their second derivative in Fig. 1C.

#### 2.3.2 Cluster analysis of a Raman image: Cell and ER

Raman images (20 × 20 µm^2^, 400 spectra) of individual cells of a total of seven cell lines (CHO‐K1, CHO‐K1‐hDAO, CHO‐S‐Humira, CHO‐S, CHO‐S/4F11, CHO‐K11D9 and CHO‐K14F10) were background subtracted (using a polynomial function of degree 3) and k‐means cluster analyzed with the following conditions: four clusters, spectral mask 400–1800 cm^−1^ (fingerprint region), Manhattan normalization mode (area under the spectral mask equal to 1), no data reduction and no pre‐transformation mode (Witec Project Plus 4.0, Witec, Germany). By this the hyperspectral image is processed to four images presenting the chemically most different four areas (clusters) and the corresponding average spectrum for each distinguished area. One cluster was assigned to ER due to higher intensity of the protein bands, one to background (which was discarded) and the remaining two clusters were more similar and merged as ”rest of the cell“ (RC). The average spectra of ER and RC of each cell, obtained from the clustering, were used in a ”principal component analysis“ (PCA) based on the second derivative of those spectra (Savitzky‐Golay Algorithm, 17 smoothing points) and the fingerprint region of 400–1800 cm^−1 ^(Unscrambler X 10.3, CAMO Software AS, USA).

### 2.4 PDI immunofluorescence staining

Cells were fixed for 10 min with 4% formaldehyde‐solution in PBS + Mg^2+^ and washed six times with PBS + Mg^2+^. After attachment to a poly‐L‐lysine coated (0.01%, Sigma) glass slide, cells were incubated with a monoclonal mouse anti‐PDI antibody (Enzo Life Sciences, 1:500 in 0.2% BSA in PBS + 0.1% saponin), washed and then stained with an Alexafluor 555 donkey anti‐mouse IgG antibody solution (molecular probes, 1:100 in 5% FCS in PBS + 0.1% saponin). After washing and mounting, images were taken with a Zeiss Axio Observer Z1 fluorescence microscope using a LCI Plan‐Neofluar 63 × 1.3 NA glycerol objective and a DsRed filter set.

## 3 Results

### 3.1 Raman microscopy differentiates CHO‐K1 host cells from two different producer cell lines

Label‐free single cell analysis methods in biotechnology are of high importance, yet still scarce. Here we evaluated the suitability of Raman microscopy for identifying a variety of CHO host and producer cell lines. Raman spectra from 300 to 3050 cm^−1 ^were acquired from CHO‐K1 host cells as well as two producer cell lines, one producing hDAO protein, a copper containing amine oxidase; the other, derived from CHO‐S, an alternative host, producing a therapeutic monoclonal antibody as one of the most important recombinant products generated in mammalian cell lines. The CHO‐K1‐hDAO cell line contained a higher concentration of Cu++ to ensure integration of copper into the active site of the enzyme. Hierarchical unsupervised clustering performed on the average Raman spectra of individual cells shows that cells from each cell line cluster together and there is a clear separation of CHO‐K1, CHO‐K1‐hDAO and CHO‐S‐Humira cells (Fig. 1A). Average spectra for each cell line are shown in Fig. 1B. Due to the similarity of spectra based on the high contribution of lipid and protein Raman characteristic bands, we also generated the first (not shown) as well as the second derivative of spectra (Fig. 1C). Second derivative spectra offer a more specific discrimination between overlapping bands and eliminate the possible effect of variable background by filtering the noise. Thereby, main differences in the spectra became apparent at wave numbers 750 (symmetric breathing of tryptophan), 1004 (phenylalanine, skeletal C‐C), 1130 (C‐C skeletal in lipids), 1304 (CH_2_ deformation in lipids and amide III and/or CH_3_, CH_2_ twisting in proteins) and 1452 (protein bands: CH_2_ bending), 1585 (C=C bending mode in proteins e.g. phenylalanine, but also present at 1580 cm^−1^ for DNA) and 1656 cm^−1^ (amide‐I of proteins and C=C of lipids), presumably due to differences in the relative protein content 29, 30, 31, 32, 33.

### 3.2 Identification of a subcellular component enriched in protein

**Figure 2:**
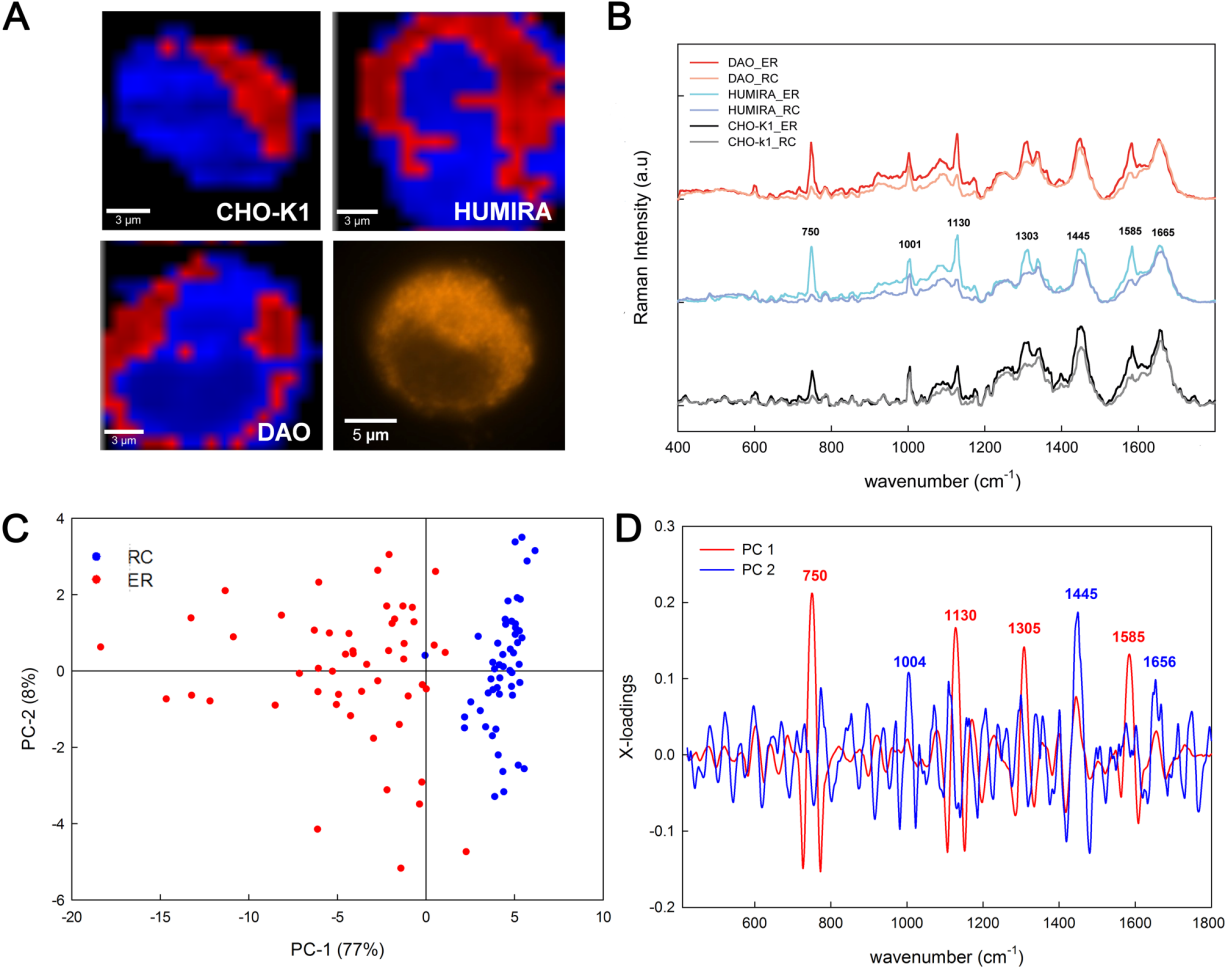
Raman microscopy differentiates a subcellular structure resembling the ER. (**A**) The individual cluster analysis (k‐means based on four clusters) of all individual cells analyzed – CHO‐K1 (*n* = 6), CHO‐K1‐hDAO (*n* = 9), CHO‐S‐Humira (*n* = 5), CHO‐S/4F11 (*n* = 7), CHO‐S (*n* = 3), CHO‐K11D9 (*n* = 11) and CHO‐K1/4F10 (*n* = 10) – differentiates a subcellular structure that resembles the endoplasmic reticulum and is highly enriched in proteins (bands at 750, 1130, 1303 and 1585 cm^−1^) (”ER“ in red) from the remaining three clusters (merged into one unique cluster afterwards) corresponding to the rest of the cell (”RC“ in blue) as visualized by the representative false color images of three representative cell lines. The bottom right image presents a CHO‐K1‐hDAO cell stained by immunofluorescence for the ER‐marker PDI, to provide a comparison of the distribution of the ER in these cells. (**B**) Corresponding average spectra for ER and RC for three representative cell lines. (**C**) The second derivative (17 smoothing points) of the average spectra of the clusters CELL and ER were subjected to PCA: Scores plot of the PC‐1 (77% explained variance) vs PC‐2 (8% explained variance) separate distinctly the clusters belonging to ER (red) and the rest of the cell (RC, blue) in all cell lines analyzed. (**D**) The X loadings indicate the positions in the spectral range responsible for the separation of the subcellular structure.

In order to picture different parts of single cells and identify protein accumulation in producer cell lines, we performed a k‐means cluster analysis 5, 34. Thereby, areas in the cells were visualized by false colors attributed to different characteristic bands that correspond to protein‐rich (putative endoplasmatic reticulum (ER) in red) or protein‐poorer (”rest of the cell“, RC in blue) regions. Representative examples of three cell lines (Fig. 2A) as well as the average spectra of ER and RC for each cell line (Fig. 2B) are shown. PCA analysis of these spectra, including those taken from all analyzed cell lines in addition to CHO‐K1, CHO‐K1‐hDAO and CHO‐S‐Humira, clearly discriminates the ER from the rest of the cell (Fig. 2C). This separation can be explained by peaks at wave numbers derived from the PC1‐loadings in Fig. 2D: 750 (symmetric breathing of aromatic tryptophan), 1130 (C‐C skeletal of acyl backbone in lipids), 1303 (CH_2_ twisting of lipids) and 1585 cm^−1^ (C=C olefinic stretch of proteins, phenylalanine) indicate that there is an enrichment of proteins and lipids in the putative ER. The X‐loadings of the rest of the cell, however, show a quite different pattern of peaks with highest signal coming from 1001 (Phenylalanine), 1445 (CH_2_ deformation band and CH_2_ bending of aliphatic amino acids) and 1665 cm^−1 ^(amide‐I vibration mode of peptide bonds) 35. Taken together, due to localization, shape and enrichment for proteins and lipids we hypothesize that this subcellular structure corresponds largely to the ER. The morphology observed strongly resembles that in an image of direct staining of ER by an antibody against protein disulfide isomerase (PDI) in CHO‐K1‐hDAO cells (Fig. 2A, bottom right).

### 3.3 PCA analysis of ER and RC discriminates a whole range of different host and producer cell lines

In order to test whether the method is generally suitable to discriminate different CHO cell lines, we included the host cell line CHO‐S as well as additional host cell subclones (CHO‐K1/F10, CHO‐K1/1D9, CHO‐S/4F11). These subclones have a phenotype of increased transient productivity relative to the parental cell line 27.

**Figure 3:**
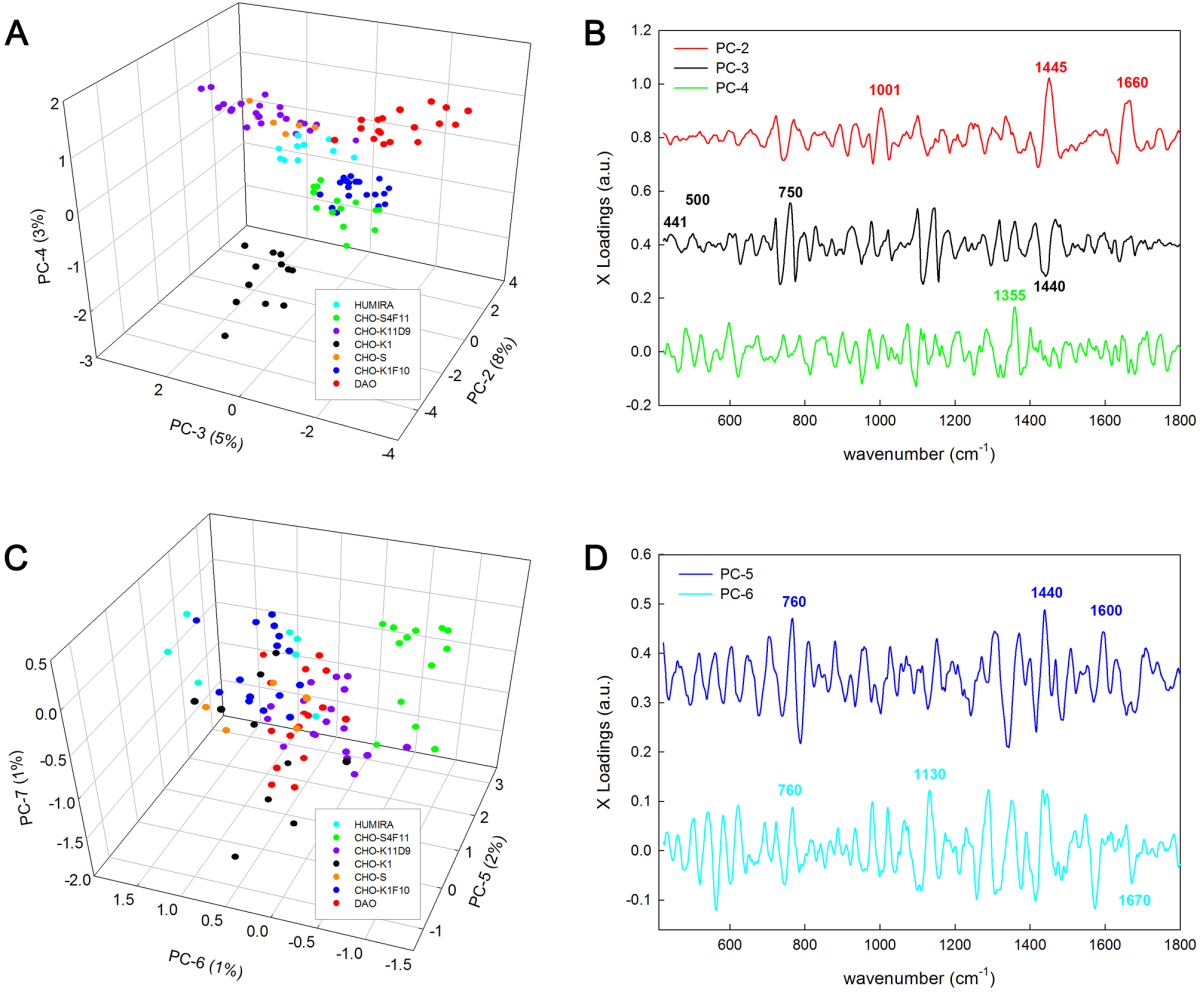
PCA of the average ER and RC spectra of a variety of CHO host and producer cell lines. For all plots, PC1 is the same as presented in Fig. 2. (**A**) PC2 against PC3 separates the host line CHO‐K1 from all other cell lines. PC3 vs PC4 differentiates the protein producer DAO and gathers the rest of cells closely in groups belonging to the same cell line. (**B**) Loading plots of PC2, PC3 and PC4. (**C**) PC5 against PC6 isolates the subclone CHO‐S4F11 with higher protein producing capacity. (**D**) Loading plots of PC5 and PC6.

The aim was to reveal spectral features that can separate closely related, but phenotypically distinct cell lines and subclones. PCA was performed with the previous ER and RC clusters obtained by k‐means clustering. After considering different possibilities, the range from 400 to 1800 cm^−1^ was selected for analysis since most of the protein bands occur in this area (Fig. 3). In PCA, the first PCs account for the highest variance in the data. Accordingly, in the present case PC1 (77% variance) separates the ER from the RC and PC2 (8% variance, based on 1445 and 1660 cm^−1^, both for lipid and protein) discriminates the CHO K1 mother cell line from all others. Interestingly, this cell line is the one with the lowest potential for productivity (all other host cell lines have a higher transient productivity 27, while the producer clones of course were selected for high productivity). In contrast, both PC3 (441 and 500 cm^−1^, possibly due to the higher copper content, and 750 cm^−1^ for protein) and PC4 separate the CHO‐K1‐hDAO from the others, while PC5 versus PC6 isolates CHO‐S/4F11, a host cell line with superior productivity. Other PCs might account for other non‐dominant sample variables. However, the other host cell lines CHO‐S, CHO‐K1/F10, CHO‐K1/1D9 were not clearly separated although cells from the same cell line are close to each other, similar to previously analyzed transcriptome data of these subclones 36. Differential transcriptome analyses there revealed an increase in ER‐specific gene expression, corresponding to relative productivity as observed here in PC1. The corresponding X‐loadings of each PC indicate the main spectral positions responsible for the differences between cell types.

## 4 Discussion and concluding remarks

Confocal Raman microscopy has been introduced recently as a promising tool for label free analysis of cells and 3D tissue cultures 37, and has been used to detect stem cell differentiation 38, tumor cells 39 and particle uptake 40. In the field of biotechnology and bioengineering, Raman spectroscopy has been used to monitor CHO cell characteristics for on‐line process monitoring 41, 42. However, to our knowledge, this is the first report on using CRM in the context of CHO cell biotechnology. In order to give proof of principle that CRM is able to differentiate CHO host and producer cells, spectra of a variety of host and producer cell lines were taken up and analyzed. Indeed, PCA and dendrograms show that the Raman spectra at specific wavelengths can differentiate not only host from producer, but also different producer cell lines, depending on the characteristics of the recombinant protein (specific amino acid composition and, in the case of DAO, higher copper content of the cells and the recombinant protein) and probably on changes in cell morphology to enable higher productivities. The pattern observed in the comparison of the different host cell types and subclones corresponds remarkably to the cluster patterns obtained during their transcriptome analysis, where CHO‐K1 also was clearly separated from all other cell types, while the CHO‐K1 subclones with increased productivity where closer to CHO‐S, with a higher starting productivity 36.

In addition, the combination of multivariate analysis and CRM reveals cellular components based on changes in the Raman spectra due to chemical differences, enabling the identification of a subcellular compartment strongly resembling the ER, clearly separated from the remaining cell 43.

The ability to distinguish between ER and the remaining cell is likely to be a major contribution towards the discrimination of high or non‐producers or even of cells that have an inherently more efficient production machinery such as the subclones used in this study, as the ER is one of the major bottlenecks encountered in high producing cell lines 44, 45, 46. Thus CRM could be used as a label free method, applicable at the single cell level during cell line development to identify promising candidates for production clones. With the experimental set up used in this study, the scanning of a single cell in the range used (400 spectra, 300–3050 cm^−1^) required 3.4 min. This time would probably be prohibitive for early clone screening during industrial cell line development campaigns. However, by selecting the most informative spectra and limiting the analysis to these, the method might be used, with proper automatization, at the stage of verification of clonality or the stage of first testing for productivity.

Finally, another important application of CRM may be as a tool to differentiate between different cell lines that produce specific products, e.g. for confirmation of identity in manufacturing.
